# Polyunsaturated fatty acids and diabetic microvascular complications: a Mendelian randomization study

**DOI:** 10.3389/fendo.2024.1406382

**Published:** 2024-08-07

**Authors:** Bingyang Liu, Ruiyan Liu, Yi Gu, Xiaoying Shen, Jianqing Zhou, Chun Luo

**Affiliations:** ^1^ Department of Critical Care Medicine, Ningbo Medical Center Lihuili Hospital, Ningbo University, Ningbo, China; ^2^ Wenzhou Medical University Renji College, Wenzhou, China; ^3^ Ningbo Institute of Innovation for Combined Medicine and Engineering, Ningbo Medical Center Lihuili Hospital, Ningbo University, Ningbo, China; ^4^ Department of Endocrinology, Ningbo Medical Center Lihuili Hospital, Ningbo University, Ningbo, China; ^5^ Department of Cardiovascular, Ningbo Medical Center Lihuili Hospital, Ningbo University, Ningbo, China

**Keywords:** diabetic microvascular complications, polyunsaturated fatty acids, Mendelian randomization, omega-6, omega-3, diabetic neuropathy, diabetic retinopathy, diabetic kidney disease

## Abstract

**Background:**

Observational studies and clinical trials have implicated polyunsaturated fatty acids (PUFAs) in potentially safeguarding against diabetic microvascular complication. Nonetheless, the causal nature of these relationships remains ambiguous due to conflicting findings across studies. This research employs Mendelian randomization (MR) to assess the causal impact of PUFAs on diabetic microvascular complications.

**Methods:**

We identified instrumental variables for PUFAs, specifically omega-3 and omega-6 fatty acids, using the UK Biobank data. Outcome data regarding diabetic microvascular complications were sourced from the FinnGen Study. Our analysis covered microvascular outcomes in both type 1 and type 2 diabetes, namely diabetic neuropathy (DN), diabetic retinopathy (DR), and diabetic kidney disease (DKD). An inverse MR analysis was conducted to examine the effect of diabetic microvascular complications on PUFAs. Sensitivity analyses were performed to validate the robustness of the results. Finally, a multivariable MR (MVMR) analysis was conducted to determine whether PUFAs have a direct influence on diabetic microvascular complications.

**Results:**

The study indicates that elevated levels of genetically predicted omega-6 fatty acids substantially reduce the risk of DN in type 2 diabetes (odds ratio (OR): 0.62, 95% confidence interval (CI): 0.47–0.82, *p* = 0.001). A protective effect against DR in type 2 diabetes is also suggested (OR: 0.75, 95% CI: 0.62–0.92, *p* = 0.005). MVMR analysis confirmed the stability of these results after adjusting for potential confounding factors. No significant effects of omega-6 fatty acids were observed on DKD in type 2 diabetes or on any complications in type 1 diabetes. By contrast, omega-3 fatty acids showed no significant causal links with any of the diabetic microvascular complications assessed.

**Conclusions:**

Our MR analysis reveals a causal link between omega-6 fatty acids and certain diabetic microvascular complications in type 2 diabetes, potentially providing novel insights for further mechanistic and clinical investigations into diabetic microvascular complications.

## 1 Introduction

Diabetes poses a significant global health challenge, affecting approximately 10% of the adult population worldwide (1). This condition markedly elevates the risk of microvascular complications, such as diabetic neuropathy (DN), diabetic retinopathy (DR), and diabetic kidney disease (DKD) (2). Significantly, >50% of those diagnosed with diabetes have DN, which can result in chronic pain, progressive sensory loss, and non-traumatic amputations (3). Additionally, 30%–40% of the patients are at risk for DR, a leading cause of blindness (4), and 30%–40% of the patients may develop DKD—the leading cause of end-stage renal disease (5). These statistics highlight the urgent need for improved prevention and treatment strategies for diabetic microvascular complications (6).

Traditionally, research on diabetic complications has focused on glucose metabolism (7). Despite intense glucose control efforts, complications continue to be prevalent, suggesting that other factors also contribute (7). Recent evidence indicates that lipids, including polyunsaturated fatty acids (PUFAs), play a crucial role in these complications (8). PUFAs mainly include omega-3 and omega-6 fatty acids. Observational studies have found that omega-6 fatty acids might protect against DN (9–11) and DR (12, 13), but their impact on DKD is uncertain (14, 15). To date, no randomized controlled trial (RCT) has evaluated the effects of omega-6 fatty acids on these conditions. By contrast, the effects of omega-3 fatty acids have been comprehensively investigated in both observational studies and RCTs, suggesting that omega-3 fatty acids may reduce the risk and severity of DN (16, 17), but their effects on DR (18–20) and DKD (14, 15, 21, 22) have been inconsistent. This emphasizes the need for research on the roles of PUFA in diabetic microvascular complications.

Mendelian randomization (MR), which employs genetic variations to simulate the random allocation inherent in RCTs, serves as an effective method for examining causal relationships in scenarios where RCTs are impractical (23). This approach precludes reverse causation by utilizing genetic predispositions that are established before disease onset (24). Although one MR study associated PUFA intake with reduced risk of DR, it did not differentiate between types of diabetes (25). This research used MR to investigate the causal links between PUFAs, specifically omega-6 and omega-3 fatty acids, and diabetic microvascular complications, including type 1 DN (T1DN), type 1 DR (T1DR), type 1 DKD (T1DKD), type 2 DN (T2DN), type 2 DR (T2DR), and type 2 DKD (T2DKD).

## 2 Methods

### 2.1 Study design

**Figure 1:**
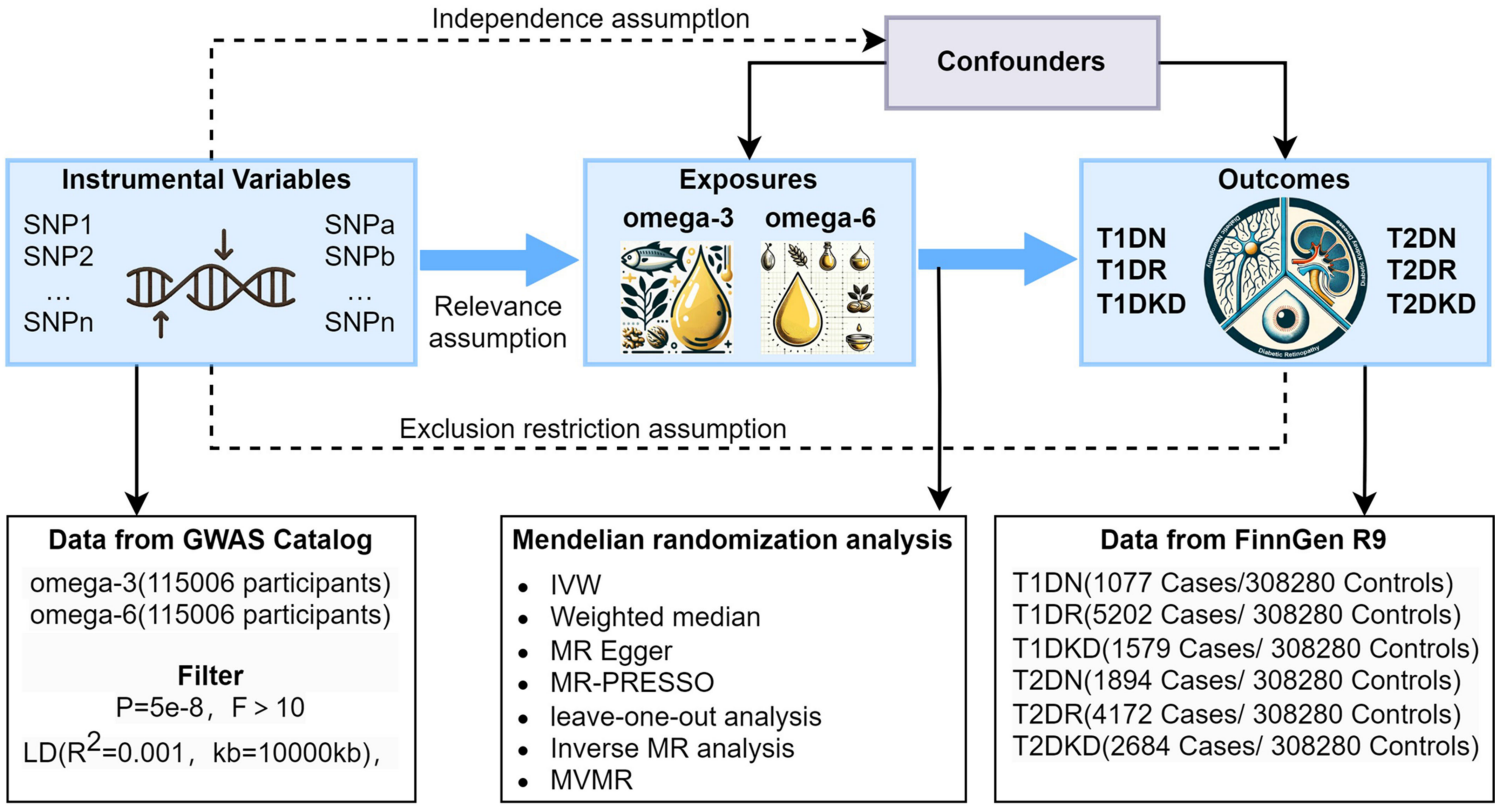
Graphical representation of the MR assumptions in a two-sample MR design, including (i) relevance; (ii) independence; and (iii) exclusion restriction. LD, linkage disequilibrium; SNPs, single nucleotide polymorphisms; IVW, inverse variance weighted; T1DN, type 1 diabetes neuropathy; T1DR, type 1 diabetes retinopathy; T1DKD, type 1 diabetic kidney disease; T2DN, type 2 diabetes neuropathy; T2DR, type 2 diabetes retinopathy; T2DKD, type 2 diabetic kidney disease.

We conducted a two-sample MR analysis using summary statistics from genome-wide association studies (GWAS) to investigate the causal relationship between PUFAs and diabetic microvascular complications. Genetic variants were used as instrumental variables (IVs) to assess the causal effects of exposure (omega-3 and omega-6) on outcomes (T1DN, T1DR, T1DKD, T2DN, T2DR, and T2DKD). Subsequently, an inverse MR analysis was performed to determine the effect of diabetic microvascular complications on PUFA levels. Given the role of lifestyle and physical conditions in the development of diabetic microvascular complications, we conducted a multivariable MR (MVMR) analysis to ascertain whether the effects of PUFAs on diabetic microvascular complications were direct or mediated by other factors. The study design is illustrated in **Figure 1**. Additionally, the causal effects of omega-3 and omega-6 fatty acids on type 1 diabetes (T1D) and type 2 diabetes (T2D) were evaluated to understand mechanisms of action. This study follows the STROBE-MR guidelines for reporting findings (26).

### 2.2 Data source

Genetic data pertaining to PUFAs were sourced from the UK Biobank, a comprehensive cohort study in the United Kingdom that recruited participants between 2006 and 2010 (27). Eligibility criteria required participants to be 40–69 years of age at recruitment (27). The UK Biobank provides a rich resource of genetic and phenotypic information (27). From this repository, we selected a random subset of >110,000 baseline EDTA plasma samples from the general population. These samples underwent analysis at Nightingale Health, where nuclear magnetic resonance and specialized software facilitated the evaluation of >200 metabolic biomarkers, including omega-6 and omega-3 fatty acids, from each blood sample (28). Among UK Biobank participants, mean concentration of omega-3 fatty acids was 0.53 mmol/L (SD: 0.22) and omega-6 fatty acids was 4.51 mmol/L (SD: 0.69).

Summary-level data on diabetic microvascular complications were obtained from the FinnGen Study, a collaborative research project in Finland (29). This project utilizes nationwide longitudinal health registry data collected from every resident in Finland since 1969, with continuous follow-up and data collection (29). This project integrates genotype data from Finnish biobanks with digital health records from national registries, to understand the genetics of various diseases in a cohort comprising 500,000 participants (29). T1DN, T1DR, T1DKD, T2DN, T2DR, and T2DKD were identified using ICD-10 codes.

The UK Biobank and FinnGen studies are based on different cohorts and geographic regions, thus minimizing the risk of sample overlap and bias. Both studies primarily consist of individuals of European ancestry, ensuring that the genetic background is similar and the associations are comparable. Standard imputation algorithms were employed in both datasets to ensure data completeness, allowing for the inclusion of all available participants. The mRnd online calculator (https://shiny.cnsgenomics.com/mRnd/) was utilized to calculate statistical power (30). With sample sizes of 310,174, 312,452, 310,964, 309,357, 313,482, and 309,859 for T2DN, T2DR, T2DKD, T1DN, T1DR, and T1DKD, respectively, the powers to detect an odds ratio (OR) of 0.7 for developing these conditions per standard deviation increase in PUFA levels (assuming genetic variants explain approximately 5% variance in PUFA levels) were 0.83, 0.99, 0.93, 0.59, 1.0, and 0.76, respectively.

In the MVMR analysis, variables such as C-reactive protein (CRP), glucose, and smoking were sourced from the UK Biobank, while interleukin 6 (IL-6) data were obtained from the study by Jing Hua Zhao et al. (31). Additionally, data for T1D and T2D used in the supplementary analyses were sourced from the Finnish database. Further details on the datasets can be found in **Supplementary Table 1**.

The UK Biobank study was approved by the North West Multi-centre Research Ethics Committee, and all participants provided written informed consent (27). The FinnGen study protocol received approval from the Ethics Committee of the Hospital District of Helsinki and Uusimaa (29). For this project, ethical approval was not required because the data were derived from the summary statistics of published GWAS and did not involve individual-level data.

### 2.3 Selection of IVs

IVs, identified as single nucleotide polymorphisms (SNPs), were selected through a rigorous screening process consistent with the core MR principles of relevance (IV is closely related to exposure), independence (IV is not related to confounders), and exclusion restriction (IV affects the outcome only through exposure) (24). Genetic variants were chosen as IVs based on their strong association with exposure, applying stringent criteria (*p* < 5 × 10^−8^ and *F*-statistic > 10) to ensure robustness. Their independence was confirmed via linkage disequilibrium analysis, adopting an *R* ^2^ threshold of <0.001 to affirm IV independence. Alignment of SNPs related to both outcome and exposure was verified to maintain methodological consistency. To identify and address potential confounding factors, we utilized the PhenoScanner database. SNPs introducing potential bias were iteratively excluded, guided by increasing *p*-values from the MR-PRESSO outlier test, until no significant outliers were detected (*p* > 0.05) in the MR-PRESSO global test. The detailed methodology, including the IV selection process, is further explicated in **Supplementary Figure 1**.

### 2.4 MR analysis and sensitivity analyses

Primary outcomes were analyzed using the inverse-variance-weighted (IVW) method. This approach is most accurate when all selected SNPs act as valid IVs (26). In the IVW method, the SNPs were weighted by the inverse of their variance. This weighting scheme provides more precise estimates by giving more weight to SNPs with smaller standard errors. The weights were derived from the GWAS summary statistics, ensuring that the contribution of each SNP to MR analysis was proportional to its precision (32). In cases of heterogeneity, we opted for a random-effects model IVW (33). We augmented the robustness of our estimates by incorporating the weighted median (WM) and MR-Egger methods. The WM method provides consistent estimates when >50% of the data comes from valid SNPs (34), whereas MR-Egger addresses potential pleiotropic effects independent of the variant-exposure association (35). Ultimately, the causal estimates were expressed as ORs with their corresponding 95% confidence intervals (CIs).MR-Egger regression was employed to assess the influence of pleiotropy among IVs. In MR-Egger regression, the intercept term serves as a test for directional pleiotropy. A non-significant intercept (*p* > 0.05) indicates the absence of systematic bias in the causal estimate due to pleiotropy. Relative symmetry in funnel plots suggests an absence of directional pleiotropy. Cochran’s Q test was conducted to assess potential heterogeneity among IVs, indicated by a non-significant result (*p* > 0.05). The “leave-one-out” analysis, which sequentially excludes one SNP at a time, further validated our results by assessing the influence of individual SNPs on the overall causal estimate (30).

### 2.5 Statistical analyses

All analyses were conducted in R version 4.3.2, using “Two-Sample MR,” “Mendelian Randomization,” “MVMR,” and “MR-PRESSO” to facilitate MR analyses. To tackle the issue of multiple testing arising from examining the relationship between two traits and six diabetes complications, Bonferroni’s correction was applied. Significance thresholds were set at *p*-value <0.004 (0.05/(2*6)) for significance, *p*-value <0.05 for nominal significance, and *p*-values between 0.004 and 0.05 for suggestive evidence.

## 3 Results

### 3.1 IVs for PUFAs and diabetic microvascular complications

Following a stringent screening process grounded in the principles of independence and exclusivity, in addition to the harmonization and elimination of palindromic SNPs with intermediate allele frequencies, we identified 36–44 SNPs for MR analysis (**Supplementary Tables 2**-**13**). The *F*-statistics for these IVs exceeded the threshold of 10, signifying their adequate strength for MR analysis and reducing the risk of bias due to weak instruments.

### 3.2 Association between genetically predicted omega-6 and diabetic microvascular complications

#### 3.2.1 Omega-6 fatty acid and DN

**Figure 2:**
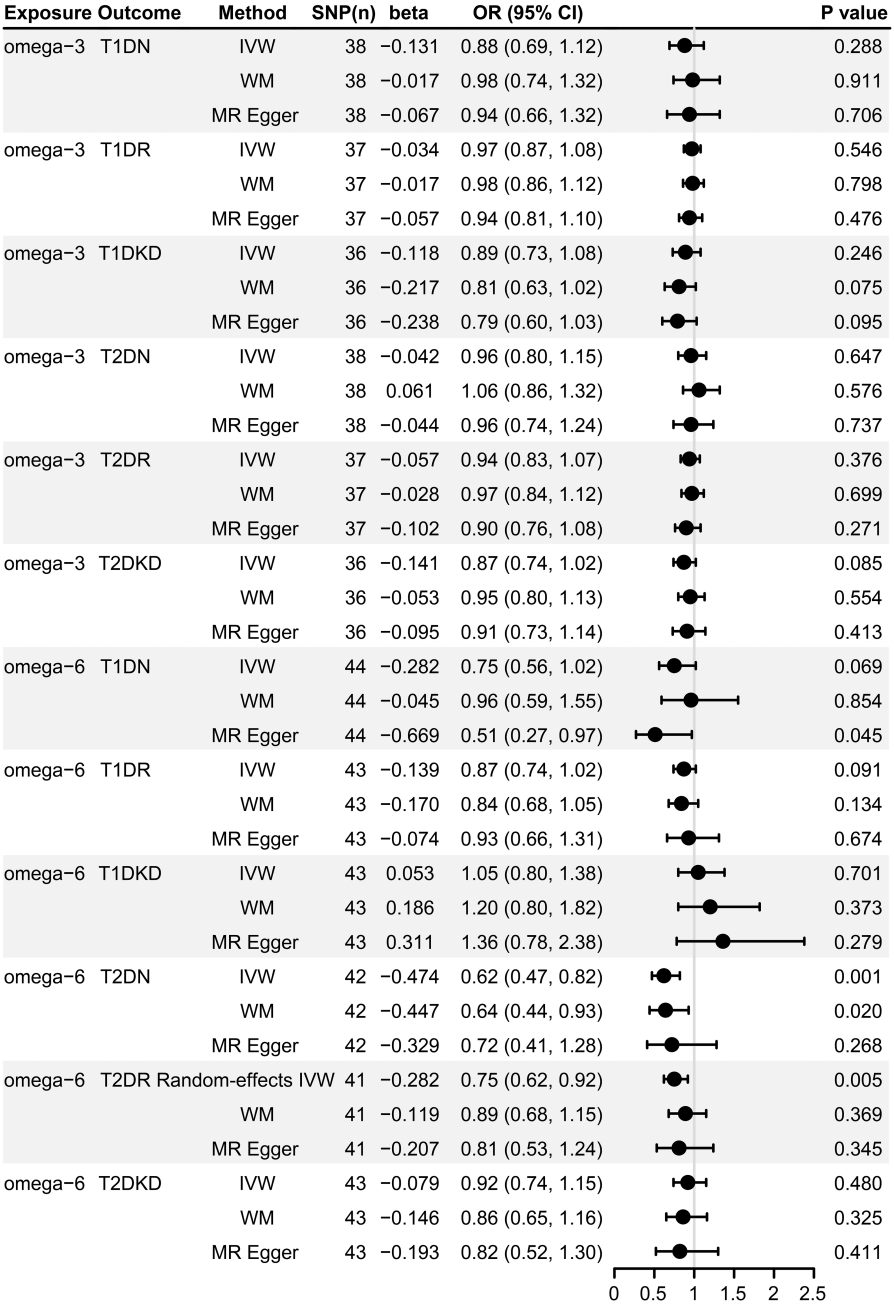
Forest plot of MR estimates of the causal associations of omega-3 and omega-6 with diabetic microvascular complications. SNP, single-nucleotide polymorphism; MR, Mendelian randomization; IVW, inverse variance weighted; WM, weighted median; T1DN, type 1 diabetes neuropathy; T1DR, type 1 diabetes retinopathy; T1DKD, type 1 diabetic kidney disease; T2DN, type 2 diabetes neuropathy; T2DR, type 2 diabetes retinopathy; T2DKD, type 2 diabetic kidney disease; CI, confidence interval; OR, odds ratio.

**Figure 3:**
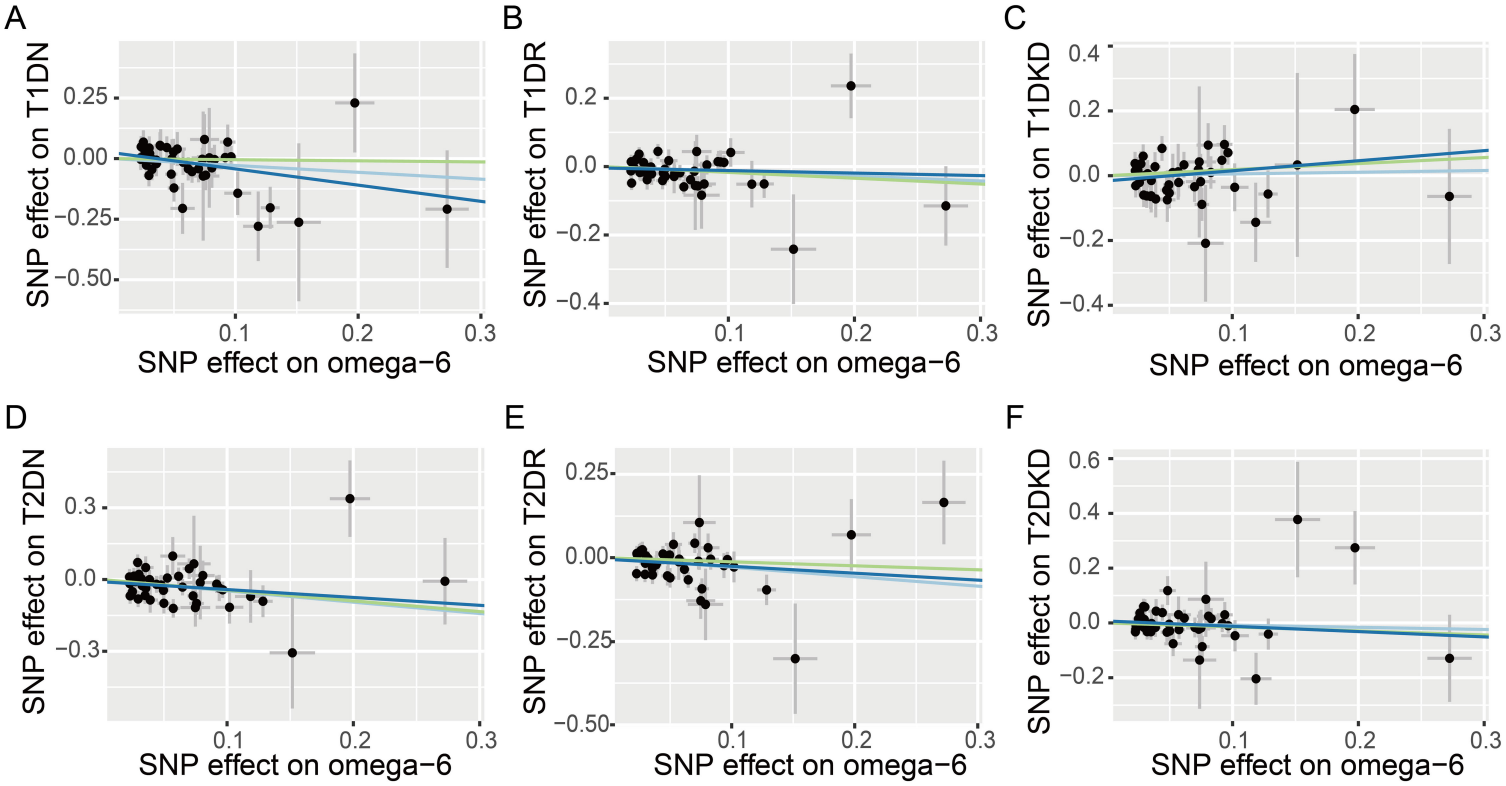
Scatter plots illustrating the effects of omega-6 polyunsaturated fatty acids on various diabetic microvascular complications, as determined by Mendelian Randomization analyses. Different colors represent the analytical methods used: light blue indicates the Inverse Variance Weighted (IVW) method, dark blue signifies MR-Egger regression, and green highlights the Weighted Median (WM) method. The conditions analyzed include: **(A)** type 1 diabetes neuropathy (T1DN); **(B)** type 1 diabetes retinopathy (T1DR); **(C)** type 1 diabetic kidney disease (T1DKD); **(D)** type 2 diabetes neuropathy (T2DN); **(E)** type 2 diabetes retinopathy (T2DR); **(F)** type 2 diabetic kidney disease (T2DKD).

**Figure 2** demonstrates that our primary IVW analysis detected a significant risk reduction for T2DN correlated with an increase of one standard deviation in genetically predicted omega-6 fatty acid levels, yielding an OR of 0.62 (95% CI: 0.47–0.82, *p* = 0.001). This translates to a 38% reduction in risk, suggesting a protective role of omega-6 fatty acids against neuropathy in patients with T2D. The WM method confirmed this association with an OR of 0.64 (95% CI: 0.44–0.93, *p* = 0.020), closely mirroring the outcomes of IVW analysis. Although MR-Egger analysis did not reach statistical significance (OR: 0.72, 95% CI: 0.41–1.28; *p* = 0.268), it exhibited a consistent direction of effect (**Figure 3**).

However, our analyses did not identify a statistically significant causal link between omega-6 levels and T1DN risk, with both IVW and WM methods resulting in *p*-values > 0.05 (**Figure 2**). However, MR-Egger estimates suggested a potential protective effect of omega-6 (OR: 0.51, 95% CI: 0.27–0.97; *p* = 0.045), albeit with caution.

#### 3.2.2 Omega-6 fatty acid and DR

The IVW method revealed a causal association between higher genetically predicted omega-6 fatty acid levels and decreased risk of T2DR, documented by an OR of 0.75 (95% CI: 0.62–0.92, *p* = 0.005). However, applying Bonferroni’s correction for multiple comparisons suggested that these findings are suggestive, and not conclusive, of a causal relationship. Additional MR methods, including WM (OR: 0.89, 95% CI: 0.68–1.15, *p* = 0.369) and MR-Egger (OR: 0.81, 95% CI: 0.53–1.24, *p* = 0.345) supported the direction of the effect but did not achieve statistical significance. This directional consistency is visually supported by **Figure 3**, indicating alignment across IVW, MR-Egger, and WM. Therefore, our research indicates a potential causal connection between omega-6 levels and reduced incidence of T2DR, awaiting further validation.

However, our analyses did not find a significant association between omega-6 fatty acids and T1DR using any MR approach (**Figure 2**, all *p*-values >0.05).

#### 3.2.3 Omega-6 fatty acid and DKD

Our study did not reveal any significant correlations between omega-6 fatty acid levels and either T1DKD or T2DKD, as evidenced by all employed analytical methods (**Figure 2**, all *p*-values > 0.05).

### 3.3 Association between genetically predicted omega-3 and diabetic microvascular complications

Our analysis did not identify any significant association between omega-3 fatty acids and microvascular complications in both types of diabetes, as determined by all analytical approaches used (**Figure 2**, all *p*-values > 0.05). Scatter plots offering a visual representation of these results can be found in **Supplementary Figure 2**.

### 3.4 Sensitivity analysis

Heterogeneity was observed in the MR analysis of omega-6 fatty acids on T2DR, with MR-Egger and IVW *p*-values of 0.034 and 0.041, respectively (**Supplementary Table 14**). Given the heterogeneity, we employed a random-effects IVW estimate to assess the causal relationship (33). This approach confirmed that higher levels of omega-6 fatty acids are significantly associated with a reduced risk of T2DR (**Figure 2**). Importantly, the MR-Egger intercept test showed no evidence of horizontal pleiotropy (*p*-values > 0.05), indicating the associations are likely genuine and not confounded by pleiotropic effects (**Supplementary Table 14**). The symmetry in funnel plots (**Supplementary Figure 3**) further supports this, indicating no directional pleiotropy or biases affecting the results. Moreover, leave-one-out sensitivity analysis reinforced the robustness of our findings, showing that no single IV disproportionately influenced overall conclusion (**Supplementary Figures 4**, **5**).

### 3.5 Inverse MR analysis

In the inverse analysis, the IVs for diabetic microvascular complications were 7–36. The IVW method revealed that T2DN and T2DKD had no effect on omega-3 or omega-6, whereas T2DR was negatively associated with omega-6 (OR: 0.98, 95% CI: 0.97–0.99, *p* = 3.71 × 10^−4^). The full details of inverse MR analysis, including results for T1DN, T1DR, and T1DKD, are provided in **Supplementary Table 15**. Analyses of heterogeneity and pleiotropy are presented in **Supplementary Table 16**.

### 3.6 MVMR analysis

**Figure 4:**
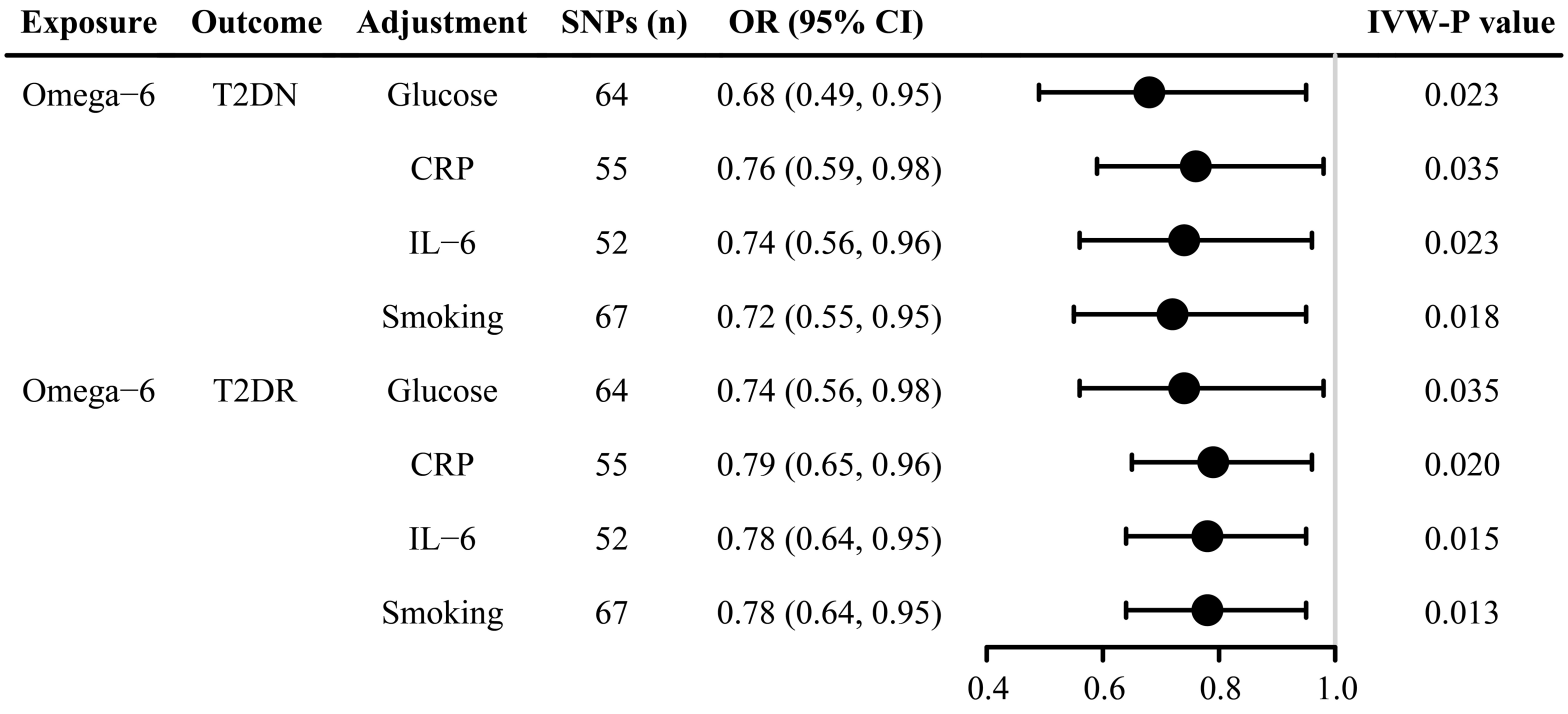
Forest plot of multivariate Mendelian randomization estimates of the causal associations after adjustment for glucose, CRP, IL-6, and smoking. SNP, single-nucleotide polymorphism; IVW, inverse variance weighted; T2DN, type 2 diabetes neuropathy; T2DR, type 2 diabetes retinopathy; CRP, C-reactive protein; IL-6, Interleukin 6; CI, confidence interval; OR, odds ratio. .

After adjusting for confounding factors, including smoking, glucose, IL-6, and CRP, omega-6 fatty acids were negatively associated with both T2DN and T2DR (**Figure 4** and **Supplementary Table 17**). Furthermore, the MVMR Egger regression showed no significant evidence of a nonzero intercept, thereby reinforcing the robustness of the MVMR analysis results (**Supplementary Table 18**).

### 3.7 Association between genetically predicted PUFAs and diabetes

We evaluated the potential causal relationships between omega-3 and omega-6 fatty acids and the risk of both T1D and T2D. The IVs were 13–41, with all *F*-statistics >10 (**Supplementary Tables 19**-**22**). The IVW analysis identified a significant reduction in the risk of T2D associated with an increase of one standard deviation in genetically predicted omega-6 fatty acid levels (OR: 0.87, 95% CI: 0.82–0.93, *p* = 3.67 × 10^−5^). This finding was corroborated by the WM method, which yielded an OR of 0.83 (95% CI: 0.76–0.91, *p* = 3.39 × 10^−5^), closely aligning with the results of IVW. Although MR-Egger analysis did not achieve statistical significance (OR: 0.90, 95% CI: 0.79–1.03, *p* = 0.146), the direction of the effect remained consistent (**Supplementary Table 23**).

By contrast, our analyses did not reveal any significant association between omega-3 fatty acids and T1D or T2D, nor between omega-6 and T1D using any MR approach (**Supplementary Table 23**, all *p*-values > 0.05).

The Q-test did not detect heterogeneity in any of the analyses. Furthermore, the MR-Egger intercept test showed no evidence of horizontal pleiotropy (all *p*-values > 0.05), indicating that these associations are likely genuine and not confounded by pleiotropic effects (**Supplementary Table 24**).

## 4 Discussion

This study employed a two-sample MR approach to investigate the causal effects of genetically determined PUFAs, specifically omega-6 and omega-3, on microvascular complications in both T1D and T2D. Our findings indicate that in T2D, elevated omega-6 levels are linked to a decreased risk of DN and DR. This relationship remained significant after adjusting for confounding factors, including smoking, glucose, IL-6, and CRP. However, omega-6 levels do not significantly influence DKD. Conversely, omega-6 levels do not seem to have a substantial effect on microvascular complications in T1D, and no significant relationships were observed between omega-3 levels and microvascular complications in either diabetes type.

Studies have focused on the effects of omega-6 fatty acids on DN, especially within the context of T2D (9–11). Aligning with these findings (9–11), our MR analysis indicates a link between omega-6 fatty acids and decreased risk of T2DN. This correlation was not observed in T1DN, implying distinct pathophysiological mechanisms underpinning DN across diabetes types. In T1DN, effective glucose management halts disease progression, whereas its influence on T2DN is less significant (36). This suggests that T2DN might be subject to additional factors, including changes in inflammation (37, 38), plasma lipid levels, and metabolic alterations (8, 39).

Previous MR studies have investigated the effects of gut microbiota and PUFAs on DR (25, 40). Kangcheng Liu et al. demonstrated a causal relationship between specific gut microbiota and DR, supporting the “gut-retina” axis concept (40). Shaojie Ren et al. found that higher levels of PUFAs, including omega-3 and omega-6, were associated with a reduced risk of DR (25). However, these studies did not distinguish between T1DR and T2DR. In contrast, our study analyzed the impact of PUFAs on both T1D and T2D and their respective retinopathies (T1DR, T2DR), providing a more comprehensive understanding of the differential effects of PUFAs on these conditions. Our findings propose a protective role of omega-6 fatty acids against T2DR and T2D, corroborating other observational studies (12, 13, 41). However, this protective effect is not observed in T1DR, possibly due to different pathogenetic mechanisms. Managing glycemic levels is crucial in controlling T1DR (42, 43), whereas T2DR may be influenced by a broader array of factors, such as inflammation (44, 45), blood pressure (46), and lipid metabolism (47, 48).

RCTs have demonstrated that diets high in omega-6 fatty acids can reduce inflammation (49, 50) Linoleic acid (LA), a primary omega-6 fatty acids, can be metabolized to arachidonic acid (AA), which is proinflammatory (51). However, only ~0.2% of dietary LA is converted to AA (52). Moreover, the levels of AA in tissues do not change with the dietary intake of LA (53). Recent studies have shown that increasing dietary LA can mitigate inflammation (50, 51). For instance, LA is negatively associated with high-sensitivity CRP (54). Pinolenic acid, another omega-6 fatty acid, demonstrated significant anti-inflammatory and anti-atherosclerotic effects by reducing the expression of inflammatory markers, such as TNF-α and IL-6 (55). Elevated levels of high-sensitivity CRP, IL-6, and TNF-α were positively associated with the risk of microvascular complications in patients with diabetes (56, 57). Therefore, omega-6 fatty acids may protect against microvascular complications by reducing inflammation.

The presence of metabolic syndrome, which includes hyperglycemia, hypertension, low high-density lipoprotein cholesterol (HDL-C), high triglycerides, and increased waist circumference, significantly elevates the risk of developing microvascular complications in patients with T2D (58). Diets rich in PUFAs have been associated with a reduced risk of metabolic syndrome (59). Our study indicates a negative association between omega-6 fatty acids and the risk of T2D, while no significant relationship was found between omega-3 fatty acids and T2D risk. These findings are consistent with results from cohort studies conducted in Asian (n = 6,393) and European (n = 14,558) populations (41). In the study, 154 metabolic biomarkers were analyzed, and 13 metabolites, including omega-6 PUFAs, were identified as being associated with a lower risk of T2D (41). However, no statistically significant association was found between omega-3 fatty acids and T2D risk (41). Additionally, a meta-analysis indicated that omega-6 PUFAs were inversely related to the risk of hypertension (60). MR analysis further demonstrated that higher serum levels of omega-6 fatty acids, particularly adrenic acid, significantly increased HDL-C levels and significantly decreased triglyceride levels (61). Omega-6 fatty acids, especially LA, were negatively associated with increase in waist circumference (62). These results further support the potential protective role of omega-6 fatty acids against microvascular complications associated with T2D.

Observational studies originating from Brazil suggest a role for omega-6 fatty acids in reducing the risk of DKD (15), yet our MR analysis, alongside observational research from China (14), has failed to establish a significant link. This discrepancy may reflect methodological divergence and genetic difference across populations. Regarding omega-3 fatty acids, although certain observational studies have posited benefits for DKD (14, 15, 21), our MR findings, complemented by results from a subsequent RCT (22), do not corroborate a significant impact. This emphasizes the critical role of robust study designs, such as MR and RCTs, in identifying causal relationships. Similarly, although observational studies from the United States (16) and a single-arm, open-label clinical trial (17) have suggested that omega-3 fatty acids potentially mitigate the risk of DN, our MR study has not found a significant association between the two.

Our investigation into the effect of omega-3 on DR did not reveal benefits, aligning with a major RCT in the UK (20). Although evidence from an earlier MR study (25) and an RCT in Spain (19) suggest a potential role for omega-3 against proliferative DR, our study did not distinguish between retinopathy subtypes. Given the lower prevalence of sight-threatening DR, including proliferative DR, which accounts for ~6% of the patients with diabetes (63), compared with the general incidence of DR among patients with diabetes estimated at 30%–40% (4), this disparity in proportions might explain why we did not observe potential benefits of omega-3 on specific DR subtypes.

Although this study offers significant insights into the influence of PUFAs on diabetic microvascular complications, several limitations merit attention. First, the MR method is inherently dependent on the assumption that selected genetic variants are accurate proxies for the exposure of interest. Despite our diligence in choosing robust instruments, the risk of bias from pleiotropic effects—where a single gene impacts multiple traits—cannot be completely disregarded, which might skew our results. We conducted sensitivity analyses to address this concern (64). Second, the genetic makeup of our study population reflects European ancestry, potentially restricting the generalizability of our findings to other ethnic groups. The interactions between genetics and environmental factors influencing diabetic complications may vary considerably across populations (65), emphasizing the need for conducting similar studies in more ethnically diverse cohorts. Estimations of PUFA intake using genetic proxies might not capture the intricate relationship between diet and disease comprehensively. Direct biomarker analysis would provide a more accurate depiction of PUFA’s influence; however, such data were not available for this research. Last, concentrating on particular PUFA subtypes enabled the identification of their specific impacts on diabetic microvascular complications. Nonetheless, this method overlooks possible synergistic or cumulative effects of different fatty acids and does not encompass broader dietary and lifestyle factors. Future research should examine the overarching influence of diet on diabetic complications, incorporating a wider array of dietary patterns and lifestyle considerations.

## 5 Conclusion

In conclusion, our study has established that genetic variants linked to omega-6 fatty acids significantly modulate the risk of T2DN and T2DR. Conversely, our analysis revealed that omega-3 fatty acids exhibit no significant correlation with diabetic microvascular complications. These findings suggest that dietary strategies emphasizing omega-6 fatty acids offer a viable preventative approach for some diabetic microvascular complications. Our results question the protective efficacy of omega-3 fatty acids in this context. Crucially, our research sets the stage for further investigations into the differential effects of omega-6 and omega-3 fatty acids, providing a foundation for future studies dedicated to uncovering mechanisms by which PUFAs influence diabetic microvascular complications.

## Data availability statement

The original contributions presented in the study are included in the article/**Supplementary Material**. Further inquiries can be directed to the corresponding author.

## Ethics statement

The UK Biobank Study was approved by the National Information Governance Board for Health and Social Care and the NHS North West Multicentre Research Ethics Committee. Ethical oversight for the FinnGen study is managed by Fimea and the HUS Coordinating Ethics Committee (HUS/990/2017), with further endorsements from THL, the Digital and Population Data Service Agency, KELA, and Statistics Finland, ensuring thorough ethical and regulatory compliance across the board. The studies were conducted in accordance with the local legislation and institutional requirements. The human samples used in this study were acquired from gifted from another research group. Written informed consent for participation was not required from the participants or the participants’ legal guardians/next of kin in accordance with the national legislation and institutional requirements.

## Author contributions

BL: Conceptualization, Funding acquisition, Investigation, Methodology, Software, Visualization, Writing – original draft. RL: Formal Analysis, Investigation, Writing – original draft. YG: Investigation, Methodology, Software, Writing – review & editing. XS: Investigation, Methodology, Writing – review & editing. JZ: Supervision, Writing – review & editing. CL: Conceptualization, Supervision, Writing – review & editing, Funding acquisition.
