# Supplementary material for: Polyunsaturated fatty acids and diabetic microvascular complications: a Mendelian randomization study
Source: Front Endocrinol (Lausanne). 2024 Aug 7;15:1406382. doi: 10.3389/fendo.2024.1406382 (PMC11335686; doi:10.3389/fendo.2024.1406382)
Supplement: Supplementary file 1 [file DataSheet_1.docx]

Supplementary Material


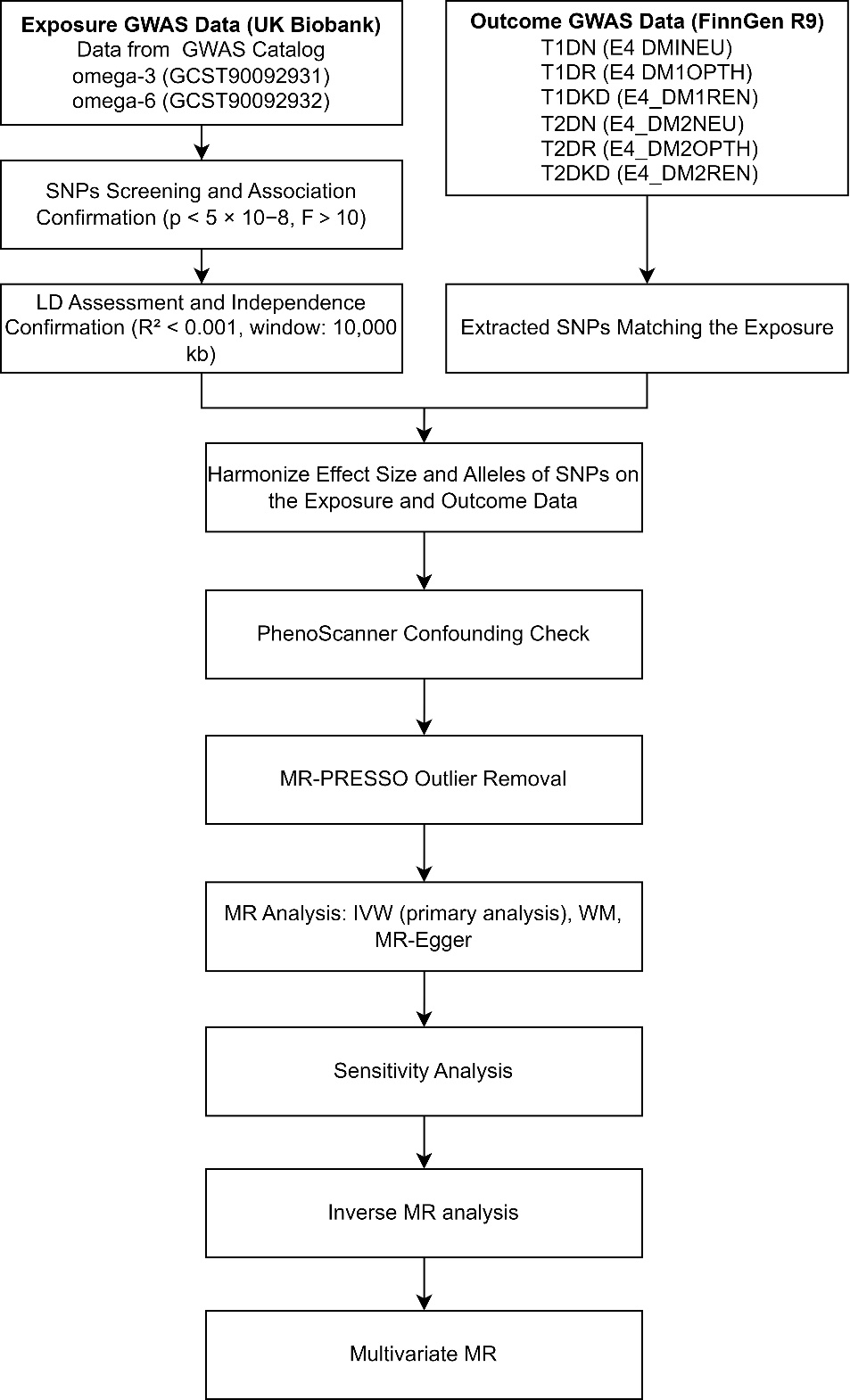


**Figure S1.** Research design and workflow diagram. SNP, single-nucleotide polymorphism; MR, Mendelian randomization; IVW, inverse variance weighted; WM, weighted median; T1DN, type 1 diabetes neuropathy; T1DR, type 1 diabetes retinopathy; T1DKD, type 1 diabetic kidney disease; T2DN, type 2 diabetes neuropathy; T2DR, type 2 diabetes retinopathy; T2DKD, type 2 diabetic kidney disease.

**
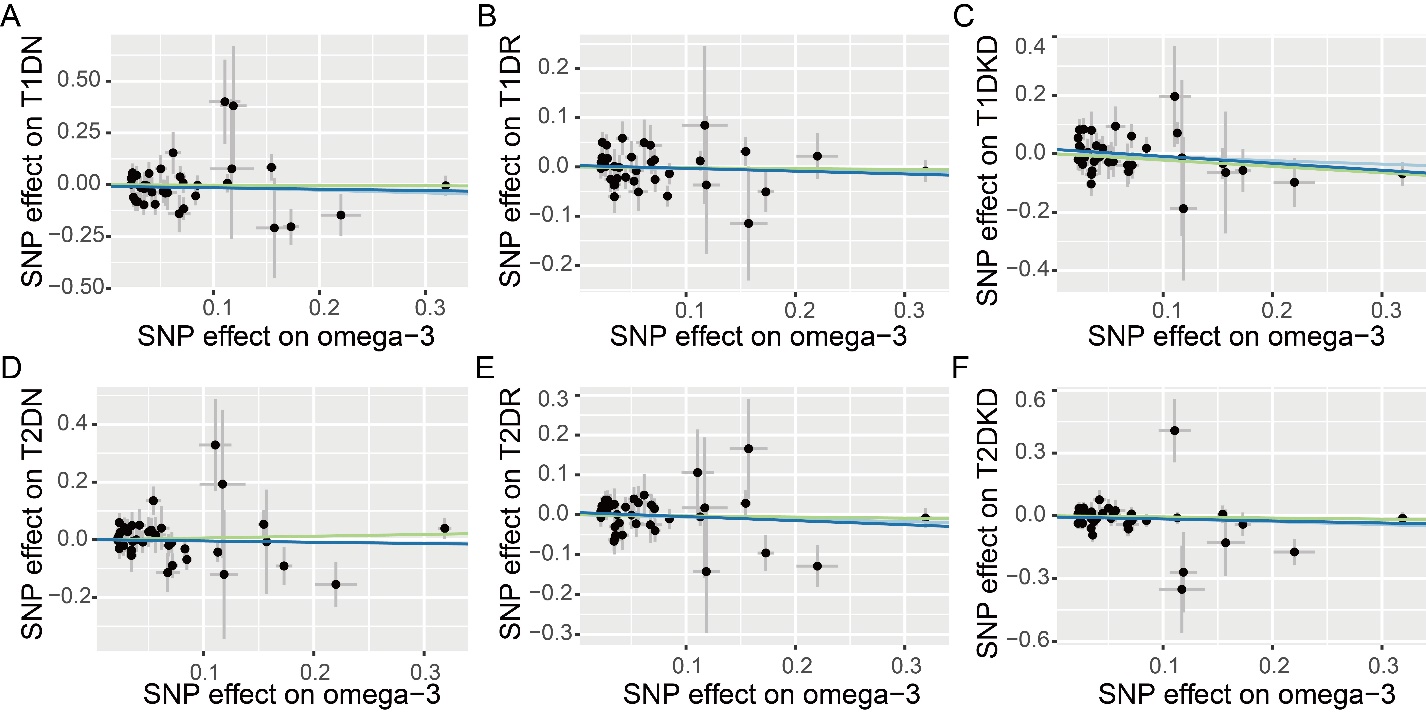
**

**Figure S2.** Scatter plots illustrating the effects of omega-3 polyunsaturated fatty acids on various diabetic microvascular complications, as determined by Mendelian Randomization analyses. Different colors represent the analytical methods used: light blue indicates the Inverse Variance Weighted (IVW) method, dark blue signifies MR-Egger regression, and green highlights the Weighted Median (WM) method. The conditions analyzed include: A) type 1 diabetes neuropathy (T1DN); B) type 1 diabetes retinopathy (T1DR); C) type 1 diabetic kidney disease (T1DKD); D) type 2 diabetes neuropathy (T2DN); E) type 2 diabetes retinopathy (T2DR); F) type 2 diabetic kidney disease (T2DKD).


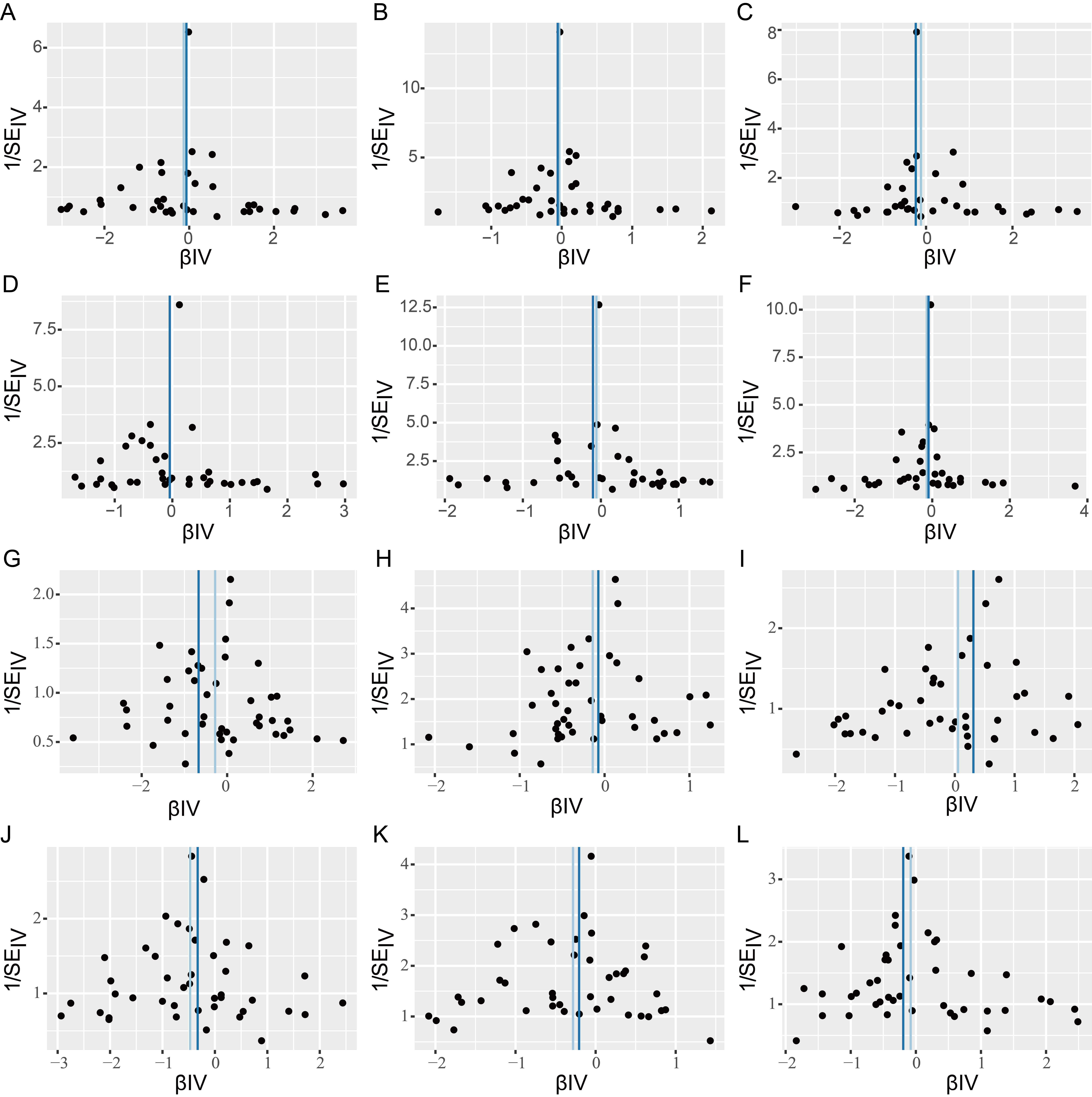


**Figure S3.** Funnel plot for assessing potential publication bias and heterogeneity in Mendelian Randomization analyses of polyunsaturated fatty acids (PUFAs) and their impact on diabetes microvascular complications. The plot displays the inverse of the standard error (indicative of study precision) on the vertical axis versus effect size estimates on the horizontal axis. The analysis encompasses the effects of omega-3 PUFAs on: A) type 1 diabetes neuropathy; B) type 1 diabetes retinopathy; C) type 1 diabetic kidney disease; D) type 2 diabetes neuropathy; E) type 2 diabetes retinopathy; F) type 2 diabetic kidney disease; and the effects of omega-6 PUFAs on: G) type 1 diabetes neuropathy; H) type 1 diabetes retinopathy; I) type 1 diabetic kidney disease; J) type 2 diabetes neuropathy; K) type 2 diabetes retinopathy; L) type 2 diabetic kidney disease.


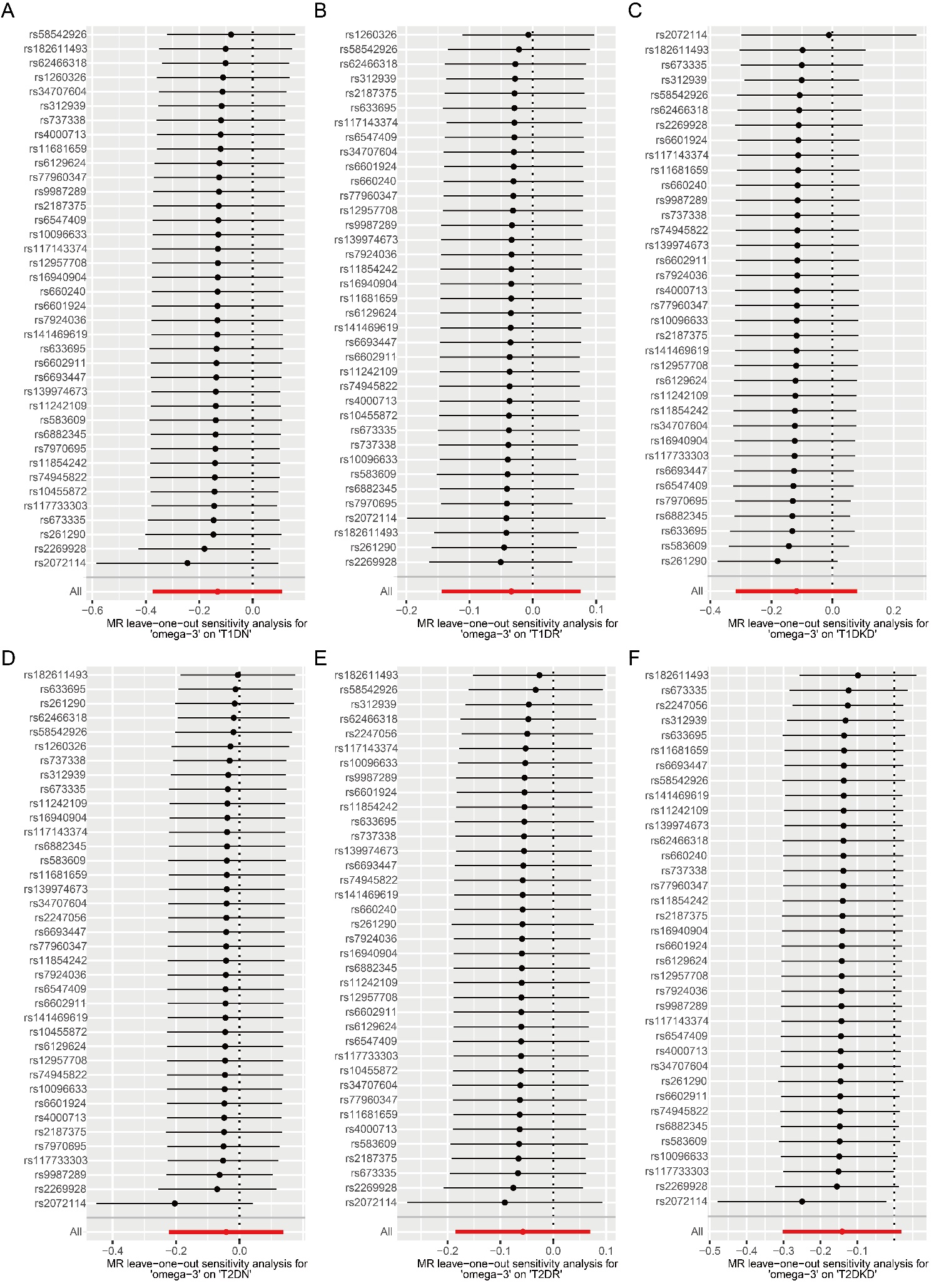


**Figure S4.** Leave-one-out sensitivity analyses in Mendelian Randomization (MR) studies assessing the impact of omega-3 fatty acids on diabetic microvascular complications. This figure illustrates the MR estimates for the relationship between omega-3 fatty acids and various diabetic microvascular complications, obtained by sequentially excluding each instrumental variable (IV) one by one. The panels show results for the effects of omega-3 on: A) type 1 diabetes neuropathy (T1DN); B) type 1 diabetes retinopathy (T1DR); C) type 1 diabetic kidney disease (T1DKD); D) type 2 diabetes neuropathy (T2DN); E) type 2 diabetes retinopathy (T2DR); F) type 2 diabetic kidney disease (T2DKD).


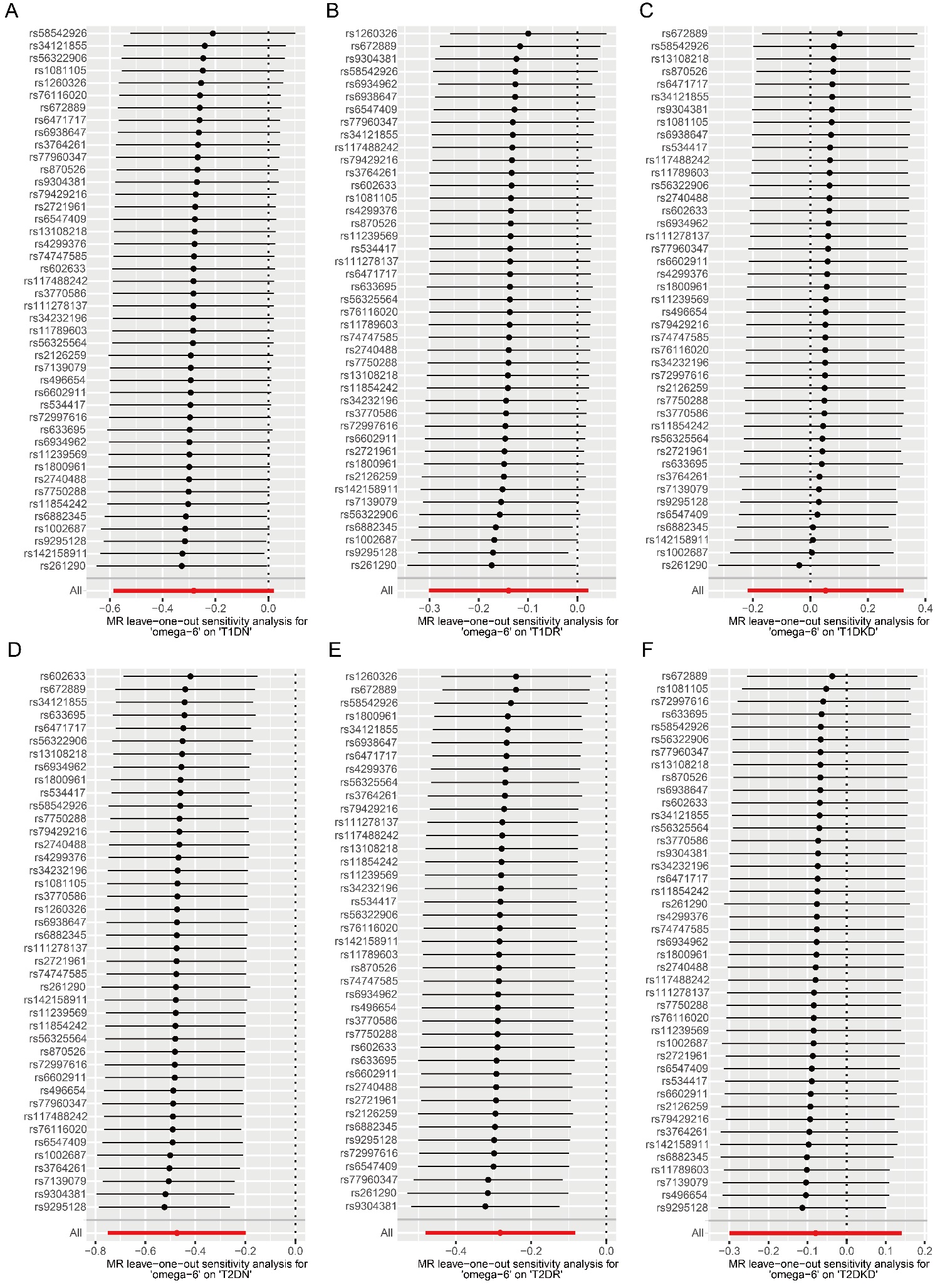


**Figure S5.** Leave-one-out sensitivity analysis for Mendelian Randomization investigations assessing the impact of omega-6 fatty acids on diabetes microvascular complications. This figure displays the MR estimates for the relationship between omega-6 fatty acids and diabetes microvascular complications, with each instrumental variable (IV) sequentially excluded one at a time. The panels illustrate the results for the effect of omega-6 on: A) type 1 diabetes neuropathy (T1DN); B) type 1 diabetes retinopathy (T1DR); C) type 1 diabetic kidney disease (T1DKD); D) type 2 diabetes neuropathy (T2DN); E) type 2 diabetes retinopathy (T2DR); F) type 2 diabetic kidney disease (T2DKD).
